# Inferring and summarizing tumor phylogenies from bulk DNA data

**DOI:** 10.1186/s13015-025-00295-5

**Published:** 2026-02-18

**Authors:** Yuanyuan Qi, Henri Schmidt, Mohammed El-Kebir

**Affiliations:** 1 Siebel School of Computing and Data Science, University of Illinois Urbana-Champaign, Urbana, IL USA; 2 Department of Computer Science, Princeton University, Princeton, NJ USA; 3 Cancer Center at Illinois, University of Illinois Urbana-Champaign, Urbana, IL USA

**Keywords:** Perfect phylogeny, Deconvolution, Maximum-agreement subtrees, Node-labeled trees, Convex optimization

## Abstract

**Background:**

Cancer phylogenies are key to understanding tumor evolution. However, due to the uncertainty in phylogenetic estimation, one typically infers many, equally-plausible phylogenies from bulk DNA sequencing data of tumors, hindering downstream analysis that relies on correct phylogenies.

**Results:**

To resolve this challenge, we introduce Sapling, a method to solve two variants of the Backbone Tree Inference from Reads problem, which seeks a small set of backbone trees on a subset of mutations that collectively summarize the space of plausible cancer phylogenies. We prove that the problems are NP-hard.

**Conclusions:**

On simulated and real data, we demonstrate that Sapling is capable of inferring high-quality backbone trees that adequately summarize the space of plausible cancer phylogenies. In addition, we demonstrate that Sapling is able to infer full-size trees with higher likelihoods than state-of-the-art methods.

## Background

Cancer results from an evolutionary process during which somatic mutations accrue in a population of cells [1]. This process results in intra-tumor heterogeneity, i.e. the presence of multiple clones with distinct sets of mutations, with important implications on cancer treatment [2]. Researchers model cancer evolution with a *phylogeny*, which is a rooted tree whose nodes correspond to clones. Cancer phylogeny methods differ from those used in species phylogenetics by explicitly modeling somatic clonal evolution while accounting for elevated error rates in single-cell sequencing and/or the presence of mixed subpopulations in bulk sequencing data [3, 4]. Cancer phylogenies are used in several downstream analyses such as immunotherapy [5] and cancer vaccines [6], metastasis analysis [7] and identification of evolutionary trajectories [8–10] (reviewed in  [3]). These downstream analyses typically require a single or a small number of phylogenies per patient. However, deconvolution of bulk DNA measurements may lead one to infer a large solution space of equally-plausible phylogenies [11].

There are three classes of methods that attempt to overcome this mismatch between the existence of large solution spaces and downstream analysis requirements. First, several approaches attempt to sample a small number of high-likelihood trees [12–16]. Second, there exist several methods that attempt to summarize a given solution space of trees with one or more consensus trees [17–23]. Another approach that also belongs to this class is SubMARine, which, rather than inferring a complete tree, returns a directed acyclic graph indicating ancestral relationships in the solution space [24]. Third, there exist approaches that use repeated evolutionary trajectories inferred from patient cohorts to reduce the number of solutions per patient [8–10, 25, 26].

**Fig. 1:**
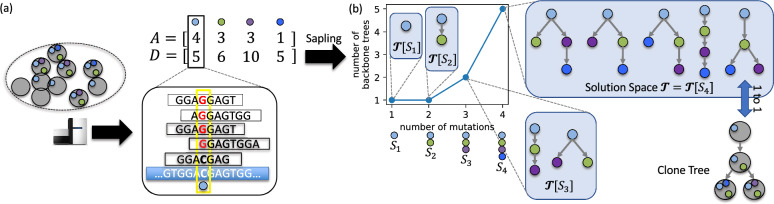
Overview of Sapling. **a** Bulk DNA sequencing, alignment and SNV calling results in matrices *A* and *D* of variant and total read counts of *n* SNVs in *m* samples. **b** Sapling is a heuristic for the Backbone Tree Inference from Reads problem, returning a small set of backbone trees for a given number $\ell$ of mutations. Here, with $\ell =3$ mutations, the solution space $\mathcal {T}$ of 5 mutation trees can be summarized with two backbone trees $\mathcal {T}[S_3]$

These three classes of methods come with their own limitations. The sampling methods, which are typically MCMC-based, exhibit great bias to certain solutions [11], and thus may not infer a representative set of solutions. The consensus methods, including methods that utilize repeated evolutionary trajectories, require an exhaustive enumeration of all plausible trees, which is impractical to obtain when the set of possible trees is large. To overcome these limitations, we introduce Sapling, a method that given read count data infers a small set of *backbone trees* on a smaller subset of mutations that collectively summarize the solution space (Fig. 1). We note that backbone trees are similar to the concept of a maximum-agreement subtree (MAST) in species phylogenetics [27], with a key distinction being that tumor phylogenies are node-labeled trees whereas species phylogenies are leaf-labeled trees. Using simulations, we show that the backbone trees returned by Sapling provide a good summary of the possible trees. We also show that Sapling is able to infer full-size trees with large numbers of mutations that are of higher quality than a current state-of-the-art tree inference method [12]. Finally, we apply Sapling to summarize non-small lung cancer solution spaces with a small number of backbone trees [28].

## Problem statement

Due to uncertainty, cancer phylogeny inference algorithms typically infer a set $\mathcal {T}$ of mutation trees from bulk sequencing data rather than a single tree. In this work, we consider trees inferred under the infinite sites assumption (ISA), meaning that each mutation is gained exactly once and never subsequently lost. We note that while this assumption does not generally hold, particularly due to copy-number loss, tumor phylogeny pipelines cluster mutations to correct for such events, yielding clusters of mutations that adhere to the ISA. Under the ISA, the solution space $\mathcal {T}$ consists of rooted trees *T* whose nodes *V*(*T*) are labeled by mutations $[n] = \{1,\ldots ,n\}$—in practice, we will view mutation clusters as individual mutations. As such, we refer to nodes and mutations interchangeably. We write $u \preceq _T v$ if node or mutation *u* occurs on the unique path from the root *r*(*T*) to node *v*—note that the relation $\preceq _T$ is reflexive, i.e., it holds that $u \preceq _T u$ for mutations *u*. We denote the set of children of a node *v* of tree *T* by $\delta _T(v)$. We denote the parent of a node $v \ne r(T)$ by $\pi _T(v)$. Our goal is to identify common features or backbone trees on a smaller set $S \subseteq [n]$ of mutations that best characterize the diversity of the solution space $\mathcal {T}$. To that end, we define backbone trees as follows.

### Definition 1

A rooted tree *T*[*S*] is a *backbone tree* of a tree *T* on mutations $S \subseteq V(T)$ provided $u \preceq _T v$ if and only if $u \preceq _{T[S]} v$ for all mutations $u,v \in S$.

Mathematically, *T* is a subdivision or expansion of *T*[*S*] such that the backbone tree *T*[*S*] is obtained from *T* by contracting nodes $V(T) \setminus S$. Rather than considering a single backbone tree on a subset *S* of mutations, we wish to identify a backbone tree set $\mathcal {T}[S]$ that collectively forms backbone trees of all trees $\mathcal {T}$ on the full mutation set [*n*].

### Definition 2

Given a set $\mathcal {T}$ of trees on *n* mutations, the corresponding *backbone tree set* $\mathcal {T}[S]$ for a subset $S \subseteq [n]$ of mutations consists of all backbone trees *T*[*S*] of all trees $T\in \mathcal {T}$.

Importantly, $|\mathcal {T}[S]|\le |\mathcal {T}|$ for all $S \subseteq [n]$. The key question is which subset $S \subseteq [n]$ of mutations provides an accurate summary of $\mathcal {T}$? Ideally, we wish to simultaneously include as many mutations as possible in *S*, i.e. maximize |*S*|, while also minimizing the number of backbone trees $|\mathcal {T}[S]|$. However, there is a tradeoff between both criteria. One can set $S=[n]$, thus maximizing |*S*|, but this would lead to as many backbone trees as there are input trees, i.e. $\mathcal {T}[S]=\mathcal {T}$, which does not provide a summary of $\mathcal {T}$. On the other hand, setting *S* to contain no mutations or just a single mutation would lead to a backbone tree set consisting of a single backbone tree composed of at most one mutation; thus while minimizing $|\mathcal {T}[S]| = 1$, this does not provide any useful information that is particular to $\mathcal {T}$. To model this tradeoff, we formulate the following two problem statements, constraining either the number |*S*| of mutations or the number $|\mathcal {T}[S]|$ of backbone trees.

### Problem 1

*(Minimum Cardinality Backbone Trees)* Given a set $\mathcal {T}$ of trees on *n* mutations and parameter $\ell \in [n]$, find a subset $S \subseteq [n]$ of $\ell$ mutations and corresponding backbone tree set $\mathcal {T}[S]$ such that $\mathcal {T}[S]$ has minimum cardinality among all backbone tree sets induced by $\ell$ mutations.

### Problem 2

*(Maximum Mutation Backbone Trees)* Given a set $\mathcal {T}$ of trees on *n* mutations and parameter $\tau \in \mathbb {N}$, find a maximum-cardinality subset $S \subseteq [n]$ of mutations and corresponding backbone tree set $\mathcal {T}[S]$ such that $|\mathcal {T}[S]| \le \tau$.

A special version of this problem arises when $\tau =1$. In that case, we are seeking a maximum-cardinality set *S* of mutations and a single corresponding backbone tree *T*[*S*] on which all trees in $\mathcal {T}$ agree.

### Backbone tree inference and expansion from reads

In practice, we are not given the set $\mathcal {T}$ of phylogenetic trees. While there exist many methods for inferring such a set via sampling [12–15] or enumeration [29, 30], obtaining the complete set $\mathcal {T}$ of trees might be infeasible due to its sheer size [11]. Therefore, we propose to infer backbone trees directly from read count data obtained from bulk DNA sequencing of *m* samples (regional or temporal) from the same tumor. More specifically, we are given two matrices $A, D \in \mathbb {N}^{m \times n}$ where $A = [a_{p,i}]$ indicates the number of reads supporting the variant allele and $D = [d_{p,i}]$ indicates the total number of reads at each mutation locus in each sample.

To pose the problem, we are interested in computing the probability $\Pr (T \mid A,D)$ of a tree *T* given the data *A*, *D*. To compute this probability, we note that the number $a_{p,i}$ of variant read counts at mutation locus *i* in sample *p* depends on the total number $d_{p,i}$ of reads, i.e. $0 \le a_{p,i} \le d_{p,i}$, and the (latent) frequency $f_{p,i} \in [0,1]$ of mutation *i* in sample *p*. Due to bulk DNA sequencing, each sample *p* is a mixture of different tumor clones such that each clone either contains or does not contain mutation *i*; therefore the frequency $f_{p,i}$ ranges between 0 and 1 rather than being either 0 or 1. Typically, one models variant read counts $A = [a_{p,i}]$ as binomial distributions, i.e. $a_{p,i} \sim \textrm{binom}(d_{p,i}, f_{p,i})$. Letting $F = [f_{p,i}]$ be the $m \times n$ frequency matrix, and using the independence of mutations and samples, we thus have

1 $$\begin{aligned} \Pr (A \mid D, F)&= \prod _{p=1}^m\prod _{i=1}^n \left( {\begin{array}{c}d_{p,i}\\ a_{p,i}\end{array}}\right) (f_{p,i})^{a_{p,i}} (1-f_{p,i})^{d_{p,i}-a_{p,i}}. \end{aligned}$$

As discussed in [31], frequencies *F* depend on a tree *T*. That is, a tree *T* under the ISA constrains frequencies $F = [f_{p,i}]$ as

SC $$\begin{aligned} f_{p,i} \ge \sum _{j \in \delta _T(i)} f_{p,j}  &   \forall p \in [m], i \in [n]. \end{aligned}$$

This is also known as the sum condition (SC). To compute the desired probability $\Pr (T \mid A,D)$, we apply Bayes’ rule, yielding $\Pr (T \mid A,D) = [\Pr (A,D \mid T) \Pr (T)]/\Pr (A,D)$. Since we observe *A*, *D*, we have that $\Pr (A,D)$ is constant. Moreover, using a flat prior on $\Pr (T)$, we obtain $\Pr (T \mid A,D) \propto \Pr (A,D \mid T)$. We now have

2 $$\begin{aligned} \Pr (A,D \mid T)&= \int _F \Pr (A \mid D,F) \Pr (F \mid T)\, dF \end{aligned}$$

3 $$\begin{aligned}&\propto \int _F \Pr (A \mid D,F) \cdot \textbf{1}\{F,T \text { satisfy } \mathrm{(SC)}\}\, dF\end{aligned}$$

4 $$\begin{aligned}&\ge \max _F \Pr (A \mid D,F) \cdot \textbf{1}\{F,T \text { satisfy } \mathrm{(SC)}\}. \end{aligned}$$

In other words, we approximate the probability $\Pr (A,D \mid T)$ of read counts *A*, *D* given a tree *T* by seeking a frequency matrix *F* such that *F* and *T* satisfy (SC) and $\Pr (A \mid D, F)$ is maximum. This is equivalent to solving the following optimization problem.

#### Definition 3

The *log-likelihood* $\mathcal {L}(A,D \mid T)$ of a rooted tree *T* and read counts *A*, *D* equals $\max _F \mathcal {L}(A, D \mid F) \text { s.t.\ } (SC)$ where $\mathcal {L}(A, D \mid F)$ is defined as $\sum _{p=1}^m\sum _{i=1}^n \left[ {a_{p,i}}\log {f_{p,i}} + {(d_{p,i} - a_{p,i})} \log {(1-f_{p,i})} \right] .$

Computing $\mathcal {L}(A,D \mid T)$, or equivalently $-\mathcal {L}(A,D \mid T)$, requires solving a convex optimization problem subject to linear constraints, which can be solved in polynomial time (for a fixed error tolerance) using interior point methods [32]. To allow one to obtain near-maximum likelihood solutions, the user may specify the parameter $\rho \in [0,1]$ yielding the solution space $\mathcal {T}^{(\rho )}$ of trees that are most a factor of $\rho$ removed from maximum likelihood, formally defined as follows.

#### Definition 4

Given $\rho \in [0,1]$ and read counts $A,D \in \mathbb {N}^{m \times n}$, the set $\mathcal {T}^{(\rho )}$ includes all trees *T* such that $\Pr (A,D \mid T) \ge \rho \Pr (A,D \mid T^*)$ where $T^*$ is a tree on *n* mutations that maximizes $\Pr (A,D \mid T^*)$.

Thus, we have $\mathcal {T}^{(\rho _1)} \subseteq \mathcal {T}^{(\rho _2)}$ for all $0 \le \rho _1 \le \rho _2 \le 1$. Specifically, for $\rho = 0$ the set $\mathcal {T}^{(0)}$ contains all $n^{n-1}$ rooted trees on *n* mutations [33]. This leads to the following updated problem statements.

#### Problem 3

*(Minimum Cardinality Backbone Trees from Reads)* Given variant and total read counts $A,D\in \mathbb {N}^{m \times n}$ for *n* mutations in *m* samples and parameters $\ell \in [n]$ and $\rho \in [0,1]$, find a subset *S* of $\ell$ mutations and corresponding backbone tree set $\mathcal {T}^{(\rho )}[S]$ such that $\mathcal {T}^{(\rho )}[S]$ has minimum cardinality among all backbone tree sets induced by $\ell$ mutations on trees $\mathcal {T}^{(\rho )}$.

#### Problem 4

*(Maximum Mutation Backbone Trees from Reads)* Given variant and total read counts $A,D\in \mathbb {N}^{m \times n}$ for *n* mutations in *m* samples and parameters $\tau \in \mathbb {N}$ and $\rho \in [0,1]$, find a maximum-cardinality subset $S \subseteq [n]$ of mutations and backbone tree set $\mathcal {T}^{(\rho )}[S]$ such that $|\mathcal {T}^{(\rho )}[S]| \le \tau$.

## Methods

In this section, we introduce the two algorithms of Sapling. First, we introduce a heuristic to solve the two Backbone Trees from Reads problems subject to either a constraint on the number of mutations or the number of backbone trees. Second, we introduce a beam-search heuristic to quickly infer full-size trees.

### Enumerating backbone trees

Given $A = [a_{p,i}]$ and $D = [d_{p,i}]$ it is clear that $\hat{F} = [\hat{f}_{p,i}]$ where $\hat{f}_{p,i} = a_{p,i} / d_{p,i}$ is the frequency matrix that maximizes $\mathcal {L}(A, D \mid T)$ when ignoring the sum condition (SC). The question whether there exists a tree *T* satisfying (SC) for a given frequency matrix was shown to be NP-complete when *F* contains $m \ge 2$ samples [30]. This means that the two problems in Section  are NP-hard when $m\ge 2$. To see why, we can set $\rho =1$ and solve the two problems with $\ell = n$ or $\tau = n^{n-1}$, respectively. This will return one or more trees on all *n* mutations. If the likelihood $\mathcal {L}(A,D \mid T)$ of any such tree *T* equals the likelihood $\mathcal {L}(A,D \mid \hat{F})$ then there exists a tree that satisfies the sum condition with $\hat{F}$, leading to the following hardness result.

#### Theorem 1

The two Backbone Trees from Reads problems (Problems 3 and 4 are NP-hard even when $m=2$.

#### Proof

We show hardness by a polynomial-time reduction from the Perfect Phylogeny Mixture Deconvolution (PPMD) problem [11, 30, 31] of deciding whether there exists a tree *T* satisfying (SC) for a given frequency matrix $F \in [0,1]^{m \times n}$. This decision problem was shown to NP-complete when *F* contains $m \ge 2$ samples [30]. Without loss of generality, we may assume that *F* is rational (the reduction from Subset Sum presented in [30] works for rational values). Thus, we have $f_{p,i} = a_{p,i}/d_{p,i}$ where $a_{p,i}, d_{p,i} \in \mathbb {N}$ and $a_{p,i} \le d_{p,i}$ for each sample $p \in [m]$ and mutation $i \in [n]$. These entries correspond to variant read count matrix $A = [a_{p,i}]$ and total read count matrix $D = [d_{p,i}]$. This reduction takes polynomial time.

We claim that Problem 3 with read counts *A*, *D* obtained from *F* is NP-hard when $m\ge 2$, $\rho =1$ and $\ell =n$. Moreover, we claim that Problem 4 with read counts *A*, *D* obtained from *F* is NP-hard when $m\ge 2$, $\rho =1$ and $\tau =n^{n-1}$. To see why, note that solving each of the three problems will return a set $\mathcal {T}$ of trees. Let $T\in \mathcal {T}$ be one such solution tree. Clearly, *T* is a tree on *n* mutations for Problem 3 and 4 due to the constraint $\ell =n$ and $\tau =n^{n-1}$, respectively. Since *F* is the maximum likelihood estimator of the binomial proportions of *A*, *D*, we have that $\mathcal {L}(A,D \mid T) \le \mathcal {L}(A,D \mid F)$. Furthermore, the likelihood $\mathcal {L}(A,D \mid T)$ equals the likelihood $\mathcal {L}(A,D \mid F)$ if and only if *T* satisfies (SC). That is, we can verify whether this bound is tight by simply checking whether *F*, *T* satisfy (SC), which takes polynomial time. $\square$

We note that the Maximum Mutation Backbone Trees from Reads problem, where we are given an upper bound $\tau$ of backbone trees, can be solved by repeatedly solving the Minimum Cardinality Backbone Trees from Reads problem, where the number $\ell$ of mutations is fixed. That is, starting with $\ell =1$, we obtain the backbone tree set $\mathcal {T}_\ell$ and increment $\ell$ until the resulting number $|\mathcal {T}_\ell |$ of trees is greater than $\tau$. We then return $\mathcal {T}_{\ell -1}$. Since the three problems are NP-hard, we cannot efficiently solve the problems exactly and introduce heuristic algorithms in the following sections.

#### A naive approach

As a first approach, we propose to build the backbone trees iteratively. We initialize the set $\mathcal {T}_1$ with a single tree *T* containing a single mutation (can be any mutation). Then, at each iteration $k \ge 2$, given the current set $\mathcal {T}_k$ on the same subset $S_k$ of mutations, we extend each tree $T \in \mathcal {T}_k$ by adding a new mutation $i \in [n] \setminus V(T)$ at each possible location. Specifically, we may either extend *T* by adding the edge (*i*, *r*(*T*)), or for an existing node $j \in V(T)$ we insert the new edge (*j*, *i*) and distribute the original children $\delta _T(j)$ among nodes *i* and *j*. This results in a new set $\mathcal {T}_{k+1}$ of trees on mutations $S_k \cup \{i\}$. The pseudocode of this $\textsc {BigExpand}(T,i)$ procedure is given in Algorithm 1.

**Algorithm 1:**
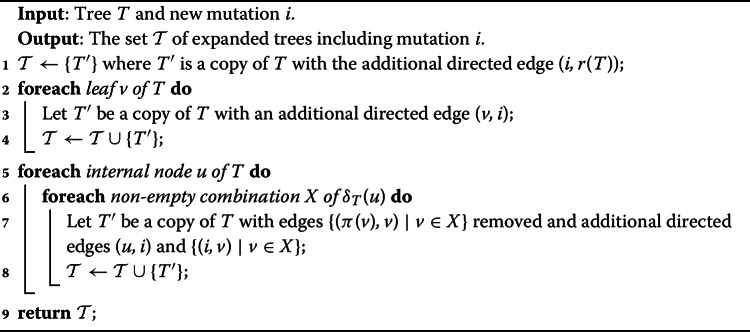
BigExpand(*T*, *i*)

In order to assess whether an expanded tree $T' \in \mathcal {T}_{k+1}$ is a good backbone tree on the mutation set $S_{k+1}=S_k\cup \{i\}$, we evaluate the likelihood $\mathcal {L}(A[S_{k+1}],D[S_{k+1}] \mid T')$. That is, we take the submatrices $A[S_{k+1}]$ of *A* and $D[S_{k+1}]$ of *D* with columns corresponding to mutations $S_{k+1}$. Computing this likelihood requires solving a convex optimization problem and, as mentioned before, this can be solved efficiently in polynomial time with a constant error tolerance [34]. We calculate $\mathcal {L}(A[S_{k+1}],D[S_{k+1}] \mid T')$ for all trees $T'$ in $\mathcal {T}_{k+1}$, retaining only those trees that have a likelihood of at least $\rho \cdot \Pr (A[S_{k+1}],D[S_{k+1}] \mid T^*)$, where $T^*$ is the maximum likelihood tree among $\mathcal {T}_{k+1}$. We terminate after iteration $\ell$, returning the set $S_{\ell }$ of mutations and backbone tree set $\mathcal {T}_{\ell }$.

However, this algorithm does not work well in practice for two key reasons. First, the order in which mutations are considered does affect the number of resulting backbone trees. One might overcome this limitation by exploring different permutations of mutations, but this becomes quickly intractable as there are *n*! permutations. Ideally, one would be able to determine a good permutation of mutations ahead of time, and only consider this single permutation when enumerating. Second, note there are $2^{|\delta _T(j)|} = O(2^n)$ possible expanded trees $T'$ when expanding a single mutation *j* of a partial tree *T*. Therefore, we need additional criteria to prune the search space.

#### Adding mutations ordered by $\hat{F}$

While we do not know the true latent frequencies of the mutations, $\hat{F} = [\hat{f}_{p,i}]$ where $\hat{f}_{p,i} = a_{p,i}/d_{p,i}$ can serve as a good estimator of the latent frequencies. As such, we propose to sort the *n* mutations in descending order based on $\sum _{p=1}^m \hat{f}_{p,i}$ and consider the mutations according to this order when enumerating backbone trees. Intuitively, this order will start by adding mutations that are closest to the root and leave the mutations that are farthest away as the last mutations to be added. We note that Orchard uses this same ordering when sampling complete trees from the solution space [12].

#### Pruning the search space

To avoid considering $O(2^n)$ possible trees when expanding a partial tree *T*, we propose to place a new mutation *i* either (i) as the new root of *T*, or (ii) as a new leaf of *T*, or (iii) split an existing edge $(\pi _T(j),j)$ of *T* inserting edges $(\pi _T(j), i)$ and (*i*, *j*). Importantly, there are only *O*(*n*) such possible expansions of a given tree *T*. The pseudocode of this $\textsc {SmallExpand}(T,i)$ procedure is given in Algorithm 2.

**Algorithm 2:**
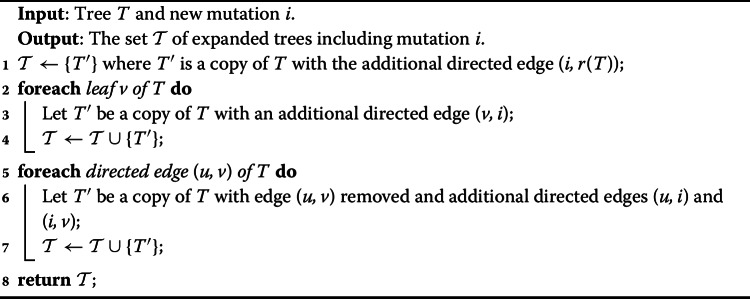
SmallExpand(*T*, *i*)

There is a theoretical justification for case (ii) of this pruning step when the *n* mutations are sorted such that $\sum _{p=1}^m \hat{f}_{p,i} \ge \sum _{p=1}^m \hat{f}_{p,j}$ for any $1 \le i < j \le n$. If the sequencing depth is large enough, $\hat{F}$ accurately reflects the latent frequencies of the mutations — i.e. $\hat{F} = [\hat{f}_{p,i}]$ is an unbiased maximum likelihood estimator if the variants read count are indeed binomially distributed. In this case, for any $\rho > 0$, each tree $T \in \mathcal {T}^{(\rho )}$ must adhere to (SC) with respect to $\hat{F}$. As such, we have that a new mutation $j > i$ cannot be a parent of a previous mutation *i*.

##### Theorem 2

Let $0 < \rho \le 1$ and let $\mathcal {T}^{(\rho )}$ be the set of corresponding trees on *n* mutations. As $d_{p,i} \rightarrow \infty$ for all *p* and *i*, then if $\sum _{p=1}^m \hat{f}_{p,i} > \sum _{p=1}^m \hat{f}_{p,j}$, it holds that $j \not \prec _T i$ for all trees $T \in \mathcal {T}^{(\rho )}$.

##### Proof

We prove this by contradiction, assuming there exists a tree $T \in \mathcal {T}^{(\rho )}$ with mutations *i*, *j* such that mutation *j* is ancestral to mutation *i* while $\sum _{p=1}^m \hat{f}_{p,i} > \sum _{p=1}^m \hat{f}_{p,j}$. Note that as $d_{p,i} \rightarrow \infty$ for all *p* and *i*, the probability distribution of $a_{p,i}$ is concentrated at $\hat{f}_{p,i}\cdot d_{p,i}$. In other words, as $[d_{p,i}] \rightarrow \infty$, we have $\Pr (\hat{F} \mid A, D) = 1$, and $\Pr (F \mid A, D) = 0$ if $F \ne \hat{F}$. Therefore, if *T* does not adhere to (SC) w.r.t. $\hat{F}$ then there must exists another matrix $F \ne \hat{F}$ such that *T* adheres to (SC) w.r.t. $F\ne \hat{F}$. However, for this $F \ne \hat{F}$ we would have $\Pr (F \mid A, D) = 0$. This in turn would imply that *T* is not in $\mathcal {T}^{(\rho )}$ for any $\rho > 0$, proving the lemma. $\square$

In practice, entries $D = [d_{p,i}]$ do not go to infinity. Therefore, we consider additional inclusion criteria beyond adding mutation *i* as a new leaf of *T*. Specifically, we allow a new mutation *i* to be a parent of an existing mutation *j* (cases (i) and (iii)). Since we do not expect the real *F* to deviate too much from $\hat{F}$, it is unlikely that mutation *i* will be assigned more than one child of mutation *j* in tree *T*, allowing us to avoid enumerating all possible redistributions of the original children of *j* among *i* and *j*. This in turn limits the number of maximum likelihood calculations. The pseudocode of the updated procedure FastBackboneEnumeration is given in Algorithm 3.

**Algorithm 3:**
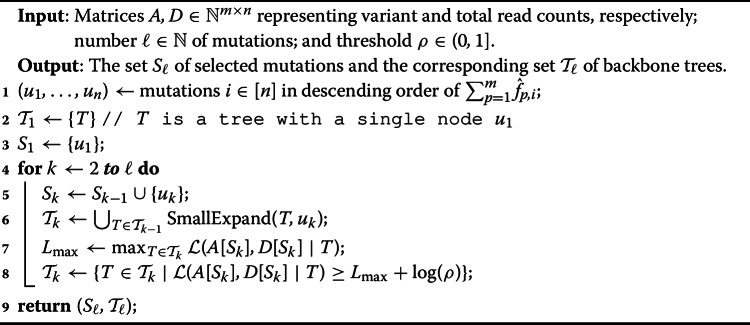
FastBackboneEnumeration(*A*, *D*, $$\ell $$, $$\rho $$)

### Inferring full trees

Setting $\ell =n$ or $\tau =\infty$ for the Minimum Cardinality Backbone Trees from Reads (Problem 3) and the Maximum Mutation Backbone Trees from Reads (Problem 4) problems, respectively, includes the problem of finding a maximum likelihood tree given reads *A*, *D*, which is NP-hard as discussed. The FastBackboneEnumeration algorithm might not scale to large number *n* of mutations. Therefore, we propose a beam-search algorithm in the following. Specifically, we take in a parameter $w\in \mathbb {N}$, specifying the beam width. The algorithm follows FastBackboneEnumeration with one exception: rather than retaining all trees that are at least a factor $\rho$ removed from maximum likelihood, we retain the top-*w* trees in terms of likelihood. For instance, specifying $w=1$ retains only a single tree at each iteration. The pseudo-code of this method called BackboneBeamEnumeration is given in Algorithm 4.

**Algorithm 4:**
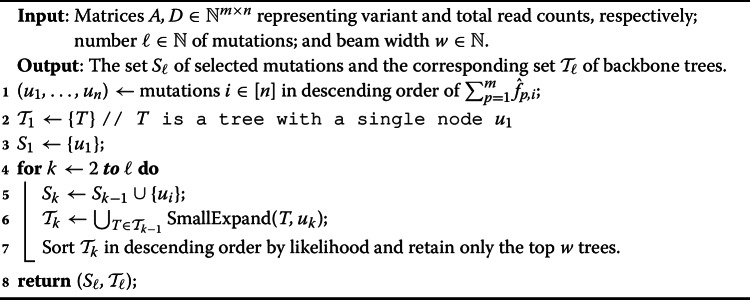
BackboneBeamEnumeration(*A*, *D*, $$\ell $$, *w*)

### Implementation details

Sapling provides a Python implementation of FastBackboneEnumeration (Algorithm 3) and BackboneBeamEnumeration (Algorithm 4). By default, Sapling uses the SmallExpand procedure (Algorithm 2) in its search, but the user has the ability to opt for BigExpand (Algorithm 1). As mentioned above, computing $-\mathcal {L}(A,D \mid T)$ is equivalent to solving a convex optimization problem with a convex objective function subject to linear constraints. Sapling uses either the Python package cvxopt [35] or fastppm [34] to optimize $-\mathcal {L}(A,D \mid T)$. If clusters of mutations are provided rather than individual mutations, Sapling takes the median depth of all mutations in the cluster as the depth of the cluster and the median depth times the average frequency $\hat{F}$ as the variant reads (rounded to the nearest integer) for the cluster and treat the cluster as a single mutation. Sapling is available at https://github.com/elkebir-group/Sapling.git under the 3-Clause BSD open source license.

## Results

In this section, we evaluate Sapling on (i) a set of small simulation instances with $n=8$ mutations and known optimal solutions, (ii) a set of larger simulation instances, and (iii) real data. All experiments were conducted on a laptop with 16 GB RAM and an Apple M1 Pro CPU. Exact command line arguments are provided in Appendix A.

### Simulation setup

To generate simulation instances with a specified number *n* of mutations and number *m* of samples, we start by randomly generating an unrooted, node-labeled tree *T* with *n* nodes/mutations using Prüfer sequences [36]. Next, we root the tree *T* uniformly at random and draw *m* samples of *n* clonal fractions from a symmetric Dirichlet distribution, yielding a frequency matrix $F = [f_{p,i}]$. For each frequency $f_{p,i}$, the total number $d_{p,i}$ of reads is drawn from a Poisson distribution with mean $\lambda =100$, simulating an average sequencing depth of 100$\times$. Finally, the variant reads $A=[a_{p,i}]$ are each drawn from a binomial distribution with $d_{p,i}$ trials and success probability $f_{p,i}$.

### Sapling identifies near-optimal backbone trees

**Fig. 2:**
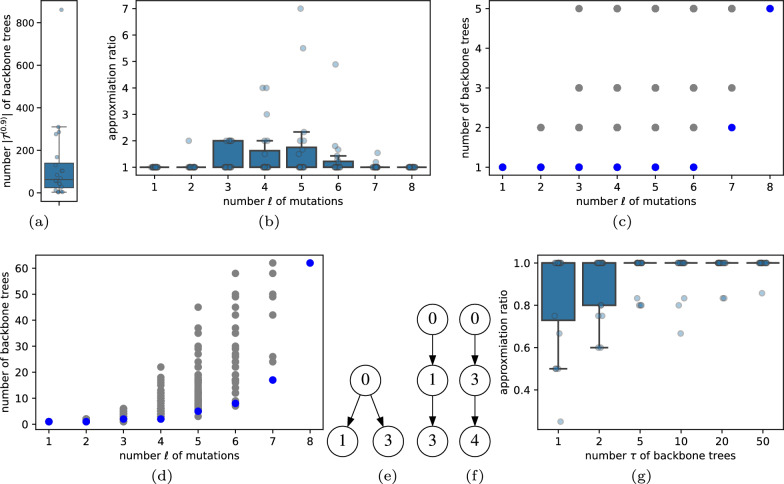
Simulations on $n=8$ mutations and $m=2$ samples. **a** The size of the set $\mathcal {T}^{(0.9)}$ of trees that have a likelihood that is at most a factor of $\rho =0.9$ away from the most likely tree. **b** Approximation ratio achieved by Sapling for varying $\ell$. **c** A simulation instance that Sapling (blue) solves to optimality for all $\ell$, with gray entries indicating subsets of mutations not considered by Sapling. **d** A simulation instance that Sapling did not solve to optimality for $\ell \in \{3,5,6\}$. **e** The two backbone trees returned by Sapling for the instance shown in **d** at $\ell =3$. **f** The optimal backbone tree of the instance in **e** determined by exhaustive enumeration at $\ell =3$. **g** Approximation ratio achieved by Sapling for varying $\tau$

We generated 20 simulated instances with $n=8$ mutations and $m=2$ samples. The small number $n=8$ of mutations allowed us to construct $\mathcal {T}^{(0.9)}$, which is the set of complete trees with a likelihood that is at most a fraction of $\rho =0.9$ away from the maximum likelihood tree. To accomplish this, we generated all $n^{n-1} = 8^7 = 2{,}097{,}152$ trees *T* and computed their likelihoods $\mathcal {L}(A,D \mid T)$, thereby constructing $\mathcal {T}^{(0.9)}$. The number $|\mathcal {T}^{(0.9)}|$ of trees ranged from 3 to 861 with a median of 62.5 trees (Fig. 2a). We ran Sapling’s FastBackboneEnumeration algorithm (Algorithm 3) with parameters $\ell \in \{1,\ldots ,8\}$ as well as $\tau \in \{1,2,5,10,20,50\}$.

We enumerated all $2^8 = 256$ subsets $S' \subseteq [n]$ of mutations, and identified the number $|\mathcal {T}^{(0.9)}[S']|$ of backbone trees for each subset $S'$ of mutations. Specifically, for each subset $S' \subseteq [n]$, we obtained $\mathcal {T}^{(0.9)}[S']$ by projecting down the ground-truth full-size trees $\mathcal {T}^{(0.9)}$ to retain only mutations $S'$. This allowed us to evaluate Sapling’s backbone trees in two ways. First, we evaluated whether each set $\mathcal {T}^{(0.9)}[S]$ of backbone trees returned by Sapling is identical to the set of backbone trees obtained by projecting down the ground-truth full-size trees $\mathcal {T}^{(0.9)}$ using the same subset *S* of mutations identified by Sapling. We found Sapling to have perfect recall (data not shown).

Second, we compared the number $|\mathcal {T}^{(0.9)}[S]|$ of backbone trees returned by Sapling for varying values of $\ell$ to the optimal number $|\mathcal {T}^{(0.9)}[S^*]|$ of backbone trees such that $|S^*|=\ell$ by computing the *approximation ratio* defined as $|\mathcal {T}^{(0.9)}[S]|/|\mathcal {T}^{(0.9)}[S^*]|$. Thus, an approximation ratio of 1 indicates that Sapling identified an optimal (minimum) set of backbone trees. We find the median approximation ratio is 1 with a maximum ratio of 7 with $|\mathcal {T}^{(0.9)}[S]| = 14$ inferred backbone trees by Sapling versus $|\mathcal {T}^{(0.9)}[S^*]| = 2$ optimal backbone trees for $\ell =5$ mutations (Fig. 2b). In Fig. 2c, we show an instance where Sapling returned optimal solutions for all $\ell$, whereas Fig. 2d shows an instance where Sapling did not return optimal solutions for all $\ell$. Specifically, for $\ell =3$ Sapling returned two backbone trees for mutations $S = \{0,1,3\}$ shown in Fig. 2e whereas there exists a different set $S^*=\{0,3,4\}$ with just a single backbone tree shown in Fig. 2f.

Similarly, we evaluate the approximation ratio when running Sapling with a specified upper bound $\tau$ of backbone trees. Specifically, let |*S*| be the number of mutations returned by Sapling and $|S^*|$ be the maximum number of mutations such that $|\mathcal {T}^{(0.9)}[S]|,|\mathcal {T}^{(0.9)}[S^*]|\le \tau$. The *approximation ratio* equals $|S|/|S^*|$, where a value of 1 indicates that Sapling returned the optimal (maximum) number of mutations and a value smaller than 1 indicates that Sapling underestimated the number of mutations. Again, we find that the median approximation ratio is 1, with a minimum ratio of 0.25 for which Sapling identified $|S|=1$ mutations versus a maximum number $|S^*|=4$ of mutations for $\tau =1$ (Fig. 2c and Table 1). In particular, for smaller $\tau$ the approximation ratio may be smaller than 1.

**Table 1:** Individual instance results for Sapling run with $\tau =1$ on simulations with $n=8$ mutations and $m=2$ samples

**Seed**	**Ground-truth** $|S^*|$	**Sapling** |*S*|	**Approximation ratio**
1	7	7	1.00
2	4	2	0.50
3	4	2	0.50
4	4	4	1.00
5	5	5	1.00
6	4	3	0.75
7	4	4	1.00
8	7	7	1.00
9	5	5	1.00
10	3	2	0.67
11	4	4	1.00
12	3	3	1.00
13	4	2	0.50
14	7	7	1.00
15	6	6	1.00
16	4	1	0.25
17	3	3	1.00
18	6	6	1.00
19	5	5	1.00
20	6	6	1.00

From left to right, the simulation identifier, the number $|S^*|$ of ground-truth mutations, the number |*S*| of mutations identified by Sapling and the approximation ratio

In summary, we find that the heuristic employed by Sapling, in the majority of cases, finds an optimal solution for these simulated instances. Moreover, we find that Sapling’s backbone trees provide an accurate summary of the ground-truth trees, with perfect recall.

### Sapling infers high-quality backbone and full trees

To demonstrate that Sapling is capable of handling large input instances, we generated 60 additional simulation instances with $m=10$ samples and $n\in \{20,50,100\}$ mutations (with 20 instances for each value of *n*). We start by discussing Sapling’s performance when inferring backbone trees with varying thresholds $\tau$. Finally, we discuss Sapling’s performance on inferring full trees, comparing it to Orchard [12].

#### Backbone trees

**Fig. 3:**
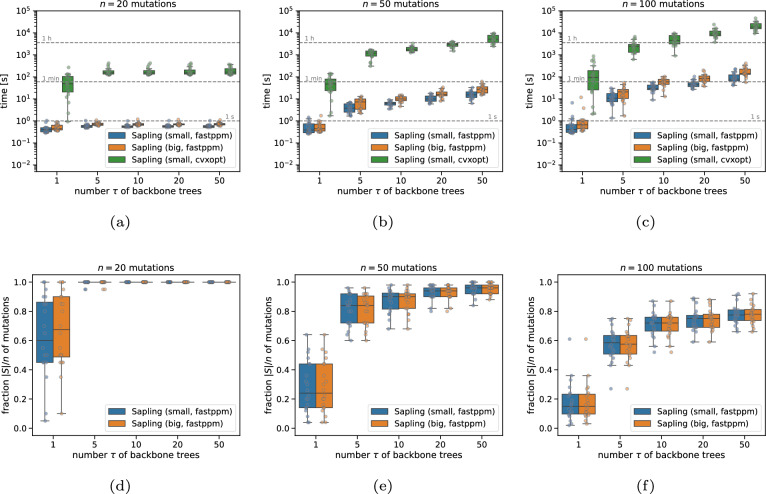
Inferring backbone trees on simulations with $n \in \{20,50,100\}$ mutations and $m=10$ samples. **a**–**c** Running time of Sapling using cvxopt vs. fastppm for varying number *n* of mutations and number $\tau$ of backbone trees. **d**–**f** The fraction of mutations included in the returned backbone tree sets

We ran FastBackboneEnumeration (Algorithm 3) as implemented in Sapling while varying $\tau \in \{1,5,10,20,50\}$ with likelihood cut-off $\rho = 0.9$. As discussed, Sapling computes the likelihood $-\mathcal {L}(A,D \mid T)$ by solving a convex optimization problem using either cvxopt [35] or fastppm [34]. We find that using fastppm leads to a speed-up of several orders of magnitude compared to cvxopt (Fig. 3a–c). While using the BigExpand (Algorithm 1) procedure results in expectedly longer running times compared to SmallExpand (Algorithm 2), it does result in slightly better performance in terms of the fraction of mutations included in the backbone tree set (Fig. 3). For example, for $n=20$ mutations and $\tau =1$, the median fraction of mutations was 0.24 for SmallExpand (median time: 0.40 s) vs. 0.27 for BigExpand (median time: 0.44 s).

#### Full trees

To compare Sapling’s full trees inferred by BackboneBeamEnumeration (Algorithm 4) to trees returned by Orchard [12] and fastBE [16], we ran all methods with varying beam widths $w \in \{1,10,50,100\}$. Specifically, we used the fastppm solver and the SmallExpand search strategy for Sapling. We ran each method in single-threaded mode.

**Fig. 4:**
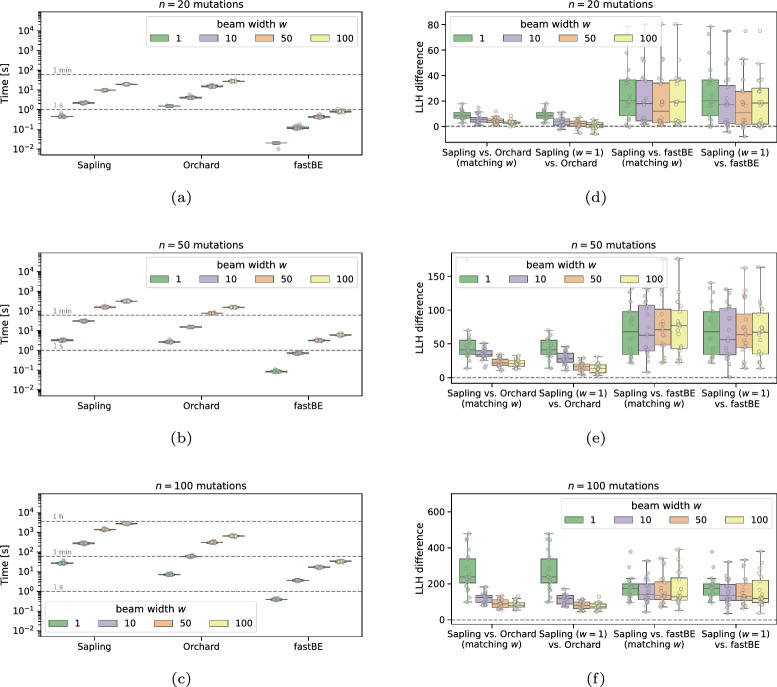
Inferring full trees on simulations with $n \in \{20,50,100\}$ mutations and $m=10$ samples. **a**–**c** Running time of Sapling vs. Orchard [14] and fastBE [16] for varying number *n* of mutations and beam widths *w*. **d**–**f** Difference in log likelihood between Sapling and Orchard for varying number *n* of mutations and beam widths *w*. Positive values indicate Sapling inferred higher likelihood solutions than Orchard

We observed that the running times of each method increased with larger beam widths *w* (Fig. 4a–c). For example, for $n=20$ mutations, the median running times range from 0.44 s ($w=1$) to 19.22 s ($w=100$) for Sapling vs. 1.51 s ($w=1$) to 27.05 s ($w=100$) for Orchard, and 0.02 s ($w=1$) to 0.80 s ($w=100$) for fastBE (Fig. 4a). We also evaluated the binomial log-likelihood of the trees inferred by Sapling and Orchard. Since Orchard uses an $L_2$ loss function on the frequencies rather than a binomial loss on the read counts, it performed much worse than Sapling (Fig. 4d–f). This was the case when using matching beam widths *w* for both methods. Strikingly, we found that Sapling with beam width $w=1$ outperformed Orchard with larger beam widths in terms of likelihood for the larger mutation regimes with $n\in \{50,100\}$ mutations, while only taking a fraction of the time (median running time of 27.91 s for Sapling $w=1$ vs. 641.12 s for Orchard $w=100$). We also observed that Sapling with beam width $w=1$ outperformed fastBE with $w=100$ in terms of likelihood for all mutation regimes. This is not surprising as fastBE approximates the log-likelihood using an $L_1$ loss.

Finally, we compared the inferred trees *T* to the ground-truth trees $T^*$ using the parent–child (PC) distance $\textrm{dist}_\textrm{PC}(T,T^*)$ and ancestor–descendant (AD) distance $\textrm{dist}_\textrm{AD}(T,T^*)$ . Specifically, the PC distance $\textrm{dist}_\textrm{PC}(T,T^*)$ equals the size of, i.e. $\textrm{dist}_\textrm{PC}(T,T^*) = |E(T) \triangle E(T^*)|/|E(T) \cup E(T^*)|$ where $\triangle$ is the symmetric difference. Letting *A*(*T*) and $A(T^*)$ be the transitive closures of *E*(*T*) and $E(T^*)$, the AD distance equals $\textrm{dist}_\textrm{AD}(T,T^*) = |A(T) \triangle A(T^*)|/|A(T) \cup A(T^*)|$ [19]. In addition, we considered the common ancestor set (CASet) and distinctly inherited set comparison (DISC) distance as introduced in [37]. All distances are between 0 and 1 where a distance of 0 is attained if and only if the two trees match. We found that the trees inferred by Sapling better matched ground-truth than Orchard’s and were of similar quality as fastBE’s across varying beam widths $w \in \{1,10,50,100\}$ and number $n\in \{20,50\}$ of mutations (Figs. S3 and S4). For example, for beam width $w=50$ and $n=20$ mutations, Sapling achieved a median PC (AD; CASet; DISC) distance of 0.54 (0.25; 0.17; 0.33) vs. 0.69 (0.32; 0.20; 0.39) for Orchard, and 0.54 (0.25; 0.16; 0.32) for fastBE (Table 2).

**Table 2:** Median PC, AD, CASet, and DISC distances for varying mutations (*n*) and beam widths (*w*)

$\boldsymbol{n}$	$\boldsymbol{w}$	**Method**	**Median distance**			
			**PC**	**AD**	**CASet**	**DISC**
20	1	Sapling	0.64	0.36	0.27	0.45
		Orchard	0.77	0.42	0.33	0.51
		fastBE	0.69	0.35	0.25	0.43
	10	Sapling	0.59	0.30	0.17	0.35
		Orchard	0.69	0.30	0.23	0.39
		fastBE	0.59	0.28	0.21	0.35
	50	Sapling	0.54	0.25	0.17	0.33
		Orchard	0.69	0.32	0.20	0.39
		fastBE	0.54	0.25	0.16	0.32
	100	Sapling	0.48	0.23	0.17	0.32
		Orchard	0.64	0.29	0.17	0.37
		fastBE	0.48	0.22	0.11	0.29
50	1	Sapling	0.89	0.54	0.39	0.63
		Orchard	0.93	0.55	0.40	0.66
		fastBE	0.89	0.53	0.38	0.63
	10	Sapling	0.89	0.53	0.38	0.62
		Orchard	0.92	0.53	0.42	0.65
		fastBE	0.89	0.52	0.38	0.62
	50	Sapling	0.89	0.53	0.38	0.61
		Orchard	0.91	0.56	0.42	0.64
		fastBE	0.87	0.51	0.35	0.61
	100	Sapling	0.89	0.51	0.38	0.59
		Orchard	0.90	0.52	0.38	0.63
		fastBE	0.86	0.51	0.35	0.61

### Sapling summarizes the solution space of real data

Finally, we applied Sapling to the TRACERx cohort of 100 non-small-cell lung cancer patients [28] using the mutation clusters reported by the authors, whose number *n* of clusters ranged from 2 to 15 and number *m* of sequencing samples ranged from 1 to 7 (details on data processing are in Appendix A.2). For each patient, we ran Sapling with parameters $\rho \in \{0.4,0.9\}$ and $\ell \in \{1,\ldots ,n\}$.

**Fig. 5:**
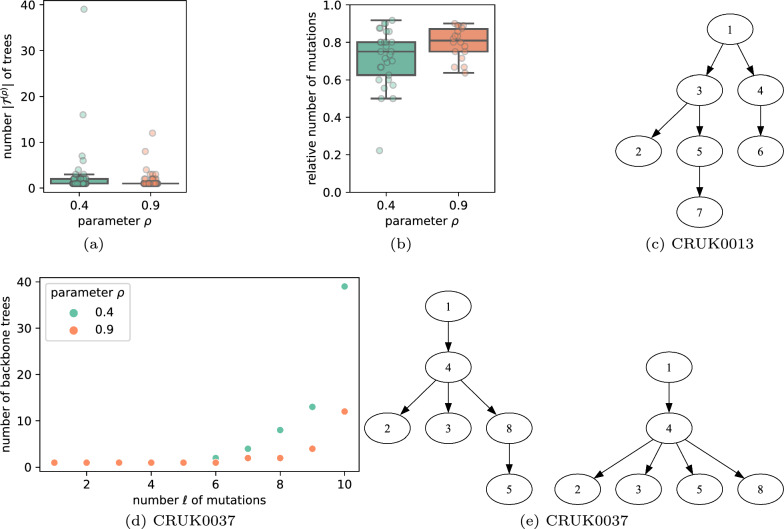
Sapling performance on TRACERx data. **a** The number $|\mathcal {T} ^{(\rho )}|$ of complete trees identified by Sapling for $\rho \in \{0.4,0.9\}$. **b** The fraction of mutation clusters in the backbone tree for $\tau =1$ (only showing patients where $|\mathcal {T} ^{(\rho )}| > 1$). **c** The single backbone tree identified by Sapling with $\tau =1,\rho =0.4$ for patient CRUK0013 (see Fig. S1, iteration $\ell =7$). **d** The number of backbone trees identified by Sapling for varying number $\ell$ of mutations for patient CRUK0037. **e** The two backbone trees identified by Sapling with $\tau =2,\rho =0.4$ for patient CRUK0037 (see Fig. S2, iteration $\ell =6$)

Setting $\ell =n$ results in Sapling enumerating the complete solution space $\mathcal {T}^{(\rho )}$ for the specified value of $\rho$, indicating the allowed deviation from maximum-likelihood. The distribution of $\mathcal {T} ^{(\rho )}$ is shown in Fig. 5a, showing that the number of trees increased with decreasing $\tau$ as expected. Specifically, there are 26 and 14 patients with at least two trees in $\mathcal {T} ^{(\rho )}$ for $\rho = 0.4$ and $\rho =0.9$, respectively.

On the other hand, when setting $\tau = 1$, Sapling seeks to identify a single backbone tree with a maximum number of mutations. In Fig. 5b, we show the fraction of mutations that are included in each individual backbone tree per patient, finding that a median fraction of 0.75 and 0.81 of mutations are included for $\rho = 0.4$ and $\rho =0.9$, respectively. We show the $\tau =1$ backbone tree identified by Sapling with $\rho =0.4$ for patient CRUK0013 with $n=9$ mutation clusters. This backbone tree spans 7 out of 9 mutation clusters, and is a proper subtree of the single consensus tree for CRUK0013 reported by the MCT algorithm [22]. For patient CRUK0037 with $n=10$ mutation clusters, restricting the number of backbone trees to $\tau =1$ results in only 5 and 6 covered mutation clusters for $\rho =0.4$ and $\rho =0.9$, respectively (Fig. 5d). With $\tau =2$ backbone trees, Sapling covers an additional mutation cluster for both values of $\rho$. We show the two backbone trees for $\rho =0.4$ in Fig. 5e, which, again, form proper subtrees of the two consensus trees reported by the MCT algorithm for this patient [22]. The backbone trees of these two patients identified by sweeping $\ell \in \{1,\ldots ,n\}$ are shown in Figs. S1 and S2.

In summary, on real data, we find that Sapling is able to quickly enumerate the complete solution space of trees and concisely summarize it with a small number of backbone trees that span a large fraction of mutations.

## Discussion

In this work, we introduced the Minimum Cardinality Backbone Trees from Reads and Maximum Mutation Backbone Trees from Reads problems, which seek to identify a set of backbone trees provided read count data. These are new problem statements, extending the concept of maximum-agreement subtree (MAST) for leaf-labeled trees [27, 38] to node-labeled trees while allowing for multiple distinct subtrees. We showed that both problems are NP-hard, and introduced a heuristic algorithm, Sapling, to solve them. Using simulations, we showed that Sapling provides a good approximate solution to both Backbone Trees from Reads problems. We also demonstrated that Sapling can be used to return full trees with higher data likelihoods than the current state-of-the-art method Orchard [12]. On real data, we ran Sapling on the TRACERx cohort of 100 lung cancer patients [28], showing that Sapling’s backbone trees adequately summarize the solution space of trees.

There are several future directions. First, we could relax the infinite sites assumption and support mutation loss. Second, for $\tau =1$, where we seek a single backbone tree with maximum number of mutations, we noted a decrease in performance. Therefore, we plan on developing a specialized algorithm for this important case, which has not been studied yet in the literature.

## Additional file

**Additional file 1.** Supplemental Materials and Methods.

## Data Availability

Sapling is available at https://github.com/elkebir-group/Sapling.git under the 3-Clause BSD open source license. The data and results reported in this manuscript are available at https://github.com/elkebir-group/Sapling-data.
