# Supplementary material for: Inferring and summarizing tumor phylogenies from bulk DNA data
Source: Algorithms Mol Biol. 2026 Feb 18;21:5. doi: 10.1186/s13015-025-00295-5 (PMC13020214; doi:10.1186/s13015-025-00295-5)
Supplement: Supplementary file 1 — Additional file 1. Supplemental Materials and Methods. [file 13015_2025_295_MOESM1_ESM.pdf]

# Appendix A Supplementary Results

## A.1 Simulations

### A.1.1 Simulations with ground truth

For the simulations with $n = 8$ mutations and $m = 2$ samples, we ran Sapling as follows.

```
for f in ../../data/sims/n8*.tsv;
do
 for ell in {1..8};
 do
  echo python \$sapling --rho 0.9 --ell $ell \
            --tau -1 -f $f \
            -o $(basename $f .tsv)_ell${ell}.tsv
 done
done
```

### A.1.2 Larger simulation instances

For the simulations with $n \in \{20, 50, 100\}$ mutations and $m = 10$ samples, we ran Sapling in three different ways. First, we ran it using SmallExpand and using `cvxopt` [35] as follows.

```
for n in 20 50 100;
do
    for filename in ../../data/sims/n${n}*.tsv;
    do
        ff=$(basename $filename .tsv)
        for tau in 1 5 10 20 50;
        do
```

```
            echo python \$sapling -f $filename \
                -o ${ff}_t${tau} -t $tau \
                -L cvxopt
        done
    done
done
```

Second, we ran Sapling using SmallExpand and using `fastppm` [34] as follows.

```
for n in 20 50 100;
do
    for filename in ../../data/sims/n${n}*.tsv;
    do
        ff=$(basename $filename .tsv)
        for tau in 1 5 10 20 50;
        do
            echo python \$sapling -f $filename \
                -o ${ff}_t${tau} -t $tau \
                -L fastppm
        done
    done
done
```

Third, we ran Sapling using BigExpand and using `fastppm` [34] as follows.

```
for n in 20 50 100;
do
    for filename in ../../data/sims/n${n}*.tsv;
    do
        ff=$(basename $filename .tsv)
        for tau in 1 5 10 20 50;
        do
```

```
            echo python \$sapling -f $filename \
                -o ${ff}_t${tau} -t $tau \
                --big_expand -L fastppm
        done
    done
done
```

### A.1.3 Full tree inference

We ran Sapling as follows to infer full trees on simulation instances with $n \in \{20, 50, 100\}$ mutations and $m = 10$ samples.

```
for n in 20 50 100;
do
    for filename in ../../data/sims/n${n}*.tsv;
    do
        ff=$(basename $filename .tsv)
        for w in 1 10 50 1000;
        do
            echo python \$sapling -f $filename \
                -o ${ff}_b$b --rho 0.9 \
                -w $w -t -1 -l -1
        done
    done
done
```

In addition, we ran Orchard as follows.

```
for f in input/*.ssm;
do
    for k in {1,10,50,100};
    do
```

```
        echo gtime -v -o $(basename $f _mutations.ssm)_k$k.time \
            python  ../../extern/orchard/bin/orchard \
            -k $k -n 1 -p $f \
            input/$(basename $f _mutations.ssm)_params.json \
            $(basename $f _mutations.ssm)_k$k.npz;
    done
done
```

We ran fastBE [16] as follows.

```
#!/usr/bin/env bash
set -euo pipefail

RESULT_DIR="results/sims_infer_full_trees_fastbe"
mkdir -p "$RESULT_DIR"

K_VALUES=(1 10 50 100)

# Loop over all frequency matrices
for freq in data/sims/*_frequency_matrix.txt; do
    # Strip suffix → get base e.g. n20_m10_s8
    base_with_path="${freq%_frequency_matrix.txt}"
    base="$(basename "$base_with_path")"

    echo "=== Processing instance: $base ==="

    variant="data/sims/${base}_variant_matrix.txt"
    total="data/sims/${base}_total_matrix.txt"
```

```
    # Loop over requested k values
    for k in "${K_VALUES[@]}"; do
        echo "  -> k = $k"

        prefix="$RESULT_DIR/${base}_k${k}"
        mkdir -p "$(dirname "$prefix")"

        #
        # 1) Run fastbe search
        #
        gtime -v fastbe search "$freq" \
            -o "$prefix" \
            -b "$k" \
            &> "${prefix}.time"

        #
        # 2) Run fastppm-cli
        #
        fastppm-cli \
            --tree "${prefix}_tree.txt" \
            --variant "$variant" \
            --total "$total" \
            -o "${prefix}_inferred_frequencies.json" \
            -f verbose \
            -l binomial
    done
done
```

## A.2 Real data analysis

We processed the original TRACERx cohort of 100 non-small-cell lung cancers [28] by extracting only those mutations that were included in tree construction in the original paper. That is, only mutations where `ActiveClusterID` does not equal 'NA' were included. We obtained the depth values from column `RegionSum`. Following the original paper, we used phylogeny and copy number corrected CCF values (in the column `PhyloCCF` to obtain variant read counts as $0.5 \cdot$ `PhyloCCF` $\cdot$ `depth`. We ran Sapling as follows.

```
#!/bin/sh
for f in ../../data/TRACERx/CRUK????.tsv
do
    ff=$(basename $f .tsv)
 nr_clusters=$(cut -f7 $f | sort -u -n | wc -l)
 for (( ell=1; ell<=nr_clusters; ell++ ))
 do
  for rho in 0.4 0.9
  do
   echo python \$sapling -f $f -l $ell \
                --rho $rho \
                -o ${ff}_ell${ell}_rho${rho}.tsv \
                --use_clusters --alt_roots
  done
 done
done
```

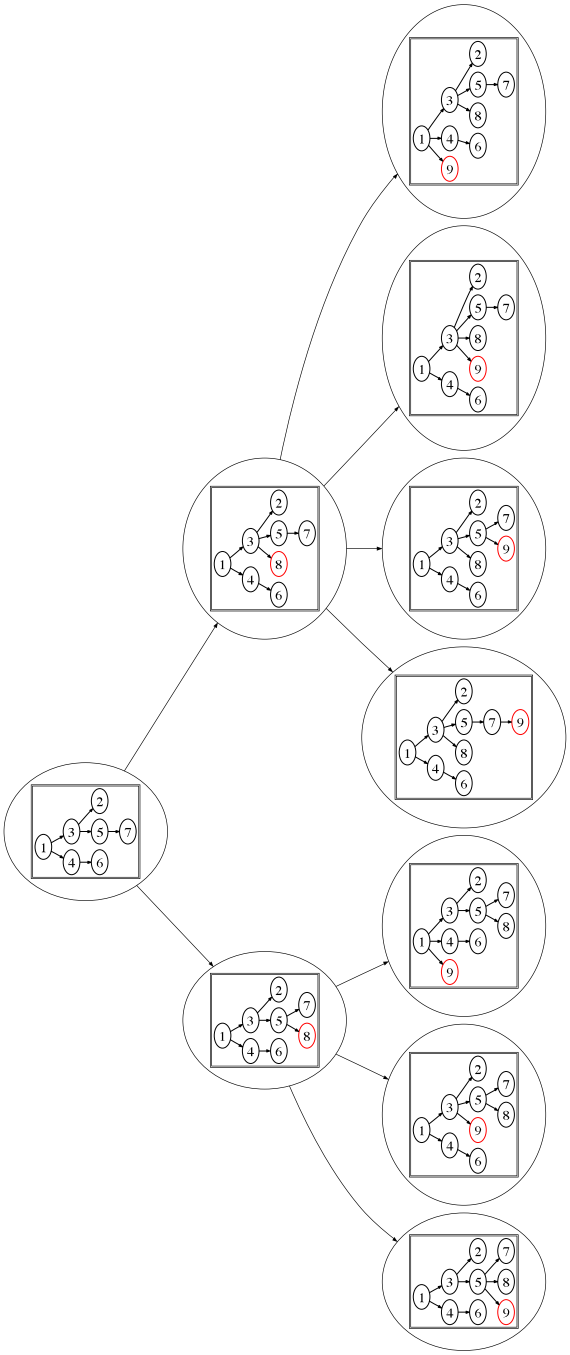

**Fig. S1**: **Results of Sapling on TRACERx patient CRUK0013.** Sapling was run iteratively starting with $\ell = 1$ and ending with $\ell = n = 9$ mutation clusters and $\rho = 0.4$. Iterations $\ell \in \{1, \ldots, 7\}$ each resulted in a single backbone tree. Note that iteration $\ell = 7$ corresponds to the single backbone tree identified in Fig 5c. Iteration $\ell = 8$ resulted in two backbone trees and the final iteration $\ell = 9$ resulted in seven complete trees. Note that the mutation cluster added in each iteration is indicated in red.

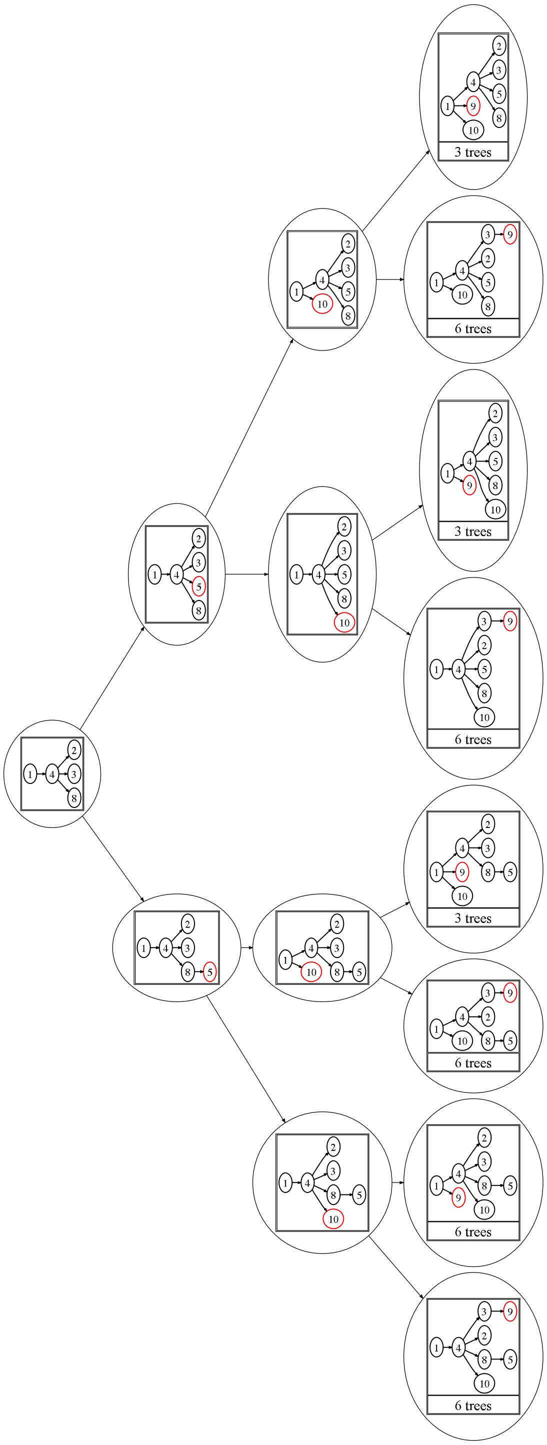


**Fig. S2**: **Results of Sapling on TRACERx patient CRUK0037.** Sapling was run iteratively starting with $\ell = 1$ and ending with $\ell = n = 10$ mutation clusters and $\rho = 0.4$. Iterations $\ell \in \{1, \ldots, 5\}$ each resulted in a single backbone tree. Iteration $\ell = 6$ resulted in two backbone trees (also shown in Fig. 5e), $\ell = 7$ resulted in 4 backbone trees, and $\ell = 8$ resulted in 8 backbone trees. Due to space constraints, backbone trees identified in iterations $\ell \in \{9, 10\}$ are not shown. Rather, we list the number of full trees summarized by each backbone tree with $\ell = 8$ mutation clusters (box). Note that the mutation cluster added in each iteration is indicated in red.

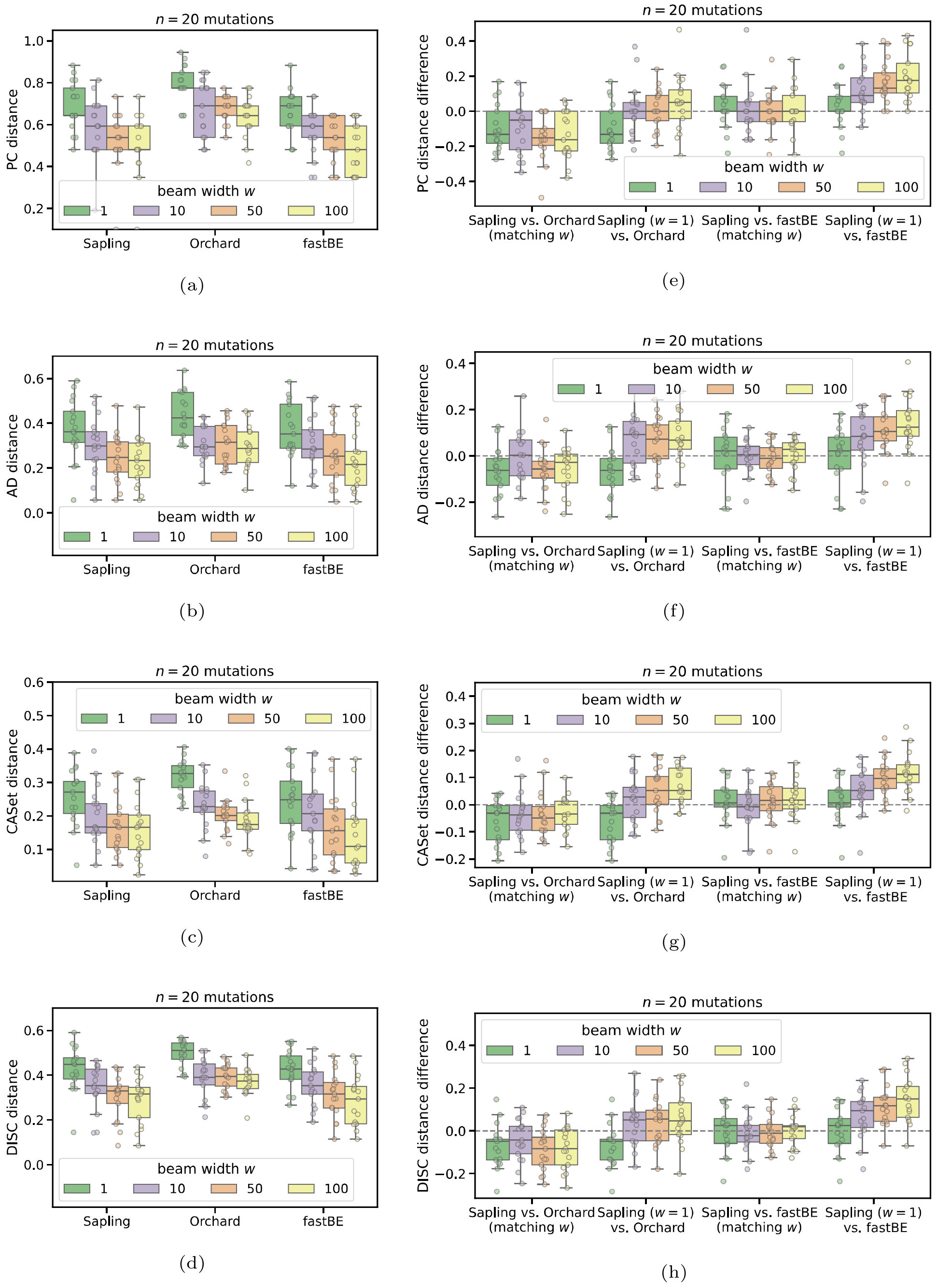


**Fig. S3**: **Inferring full trees on simulations with $n = 20$ mutations and $m = 10$ samples.** (a-d) Parent-child (PC), ancestor-descendant (AD), common ancestor set (CASet) and distinctly inherited set comparison (DISC) distances, respectively, of inferred to ground-truth trees for varying beam widths $w$. (e-f) Difference in PC, AD, CASet and DISC distances, respectively, between Sapling vs. Orchard and fastBE for matching instances and varying beam widths $w$. Negative values indicate Sapling inferred trees are closer to ground truth than the competing method.

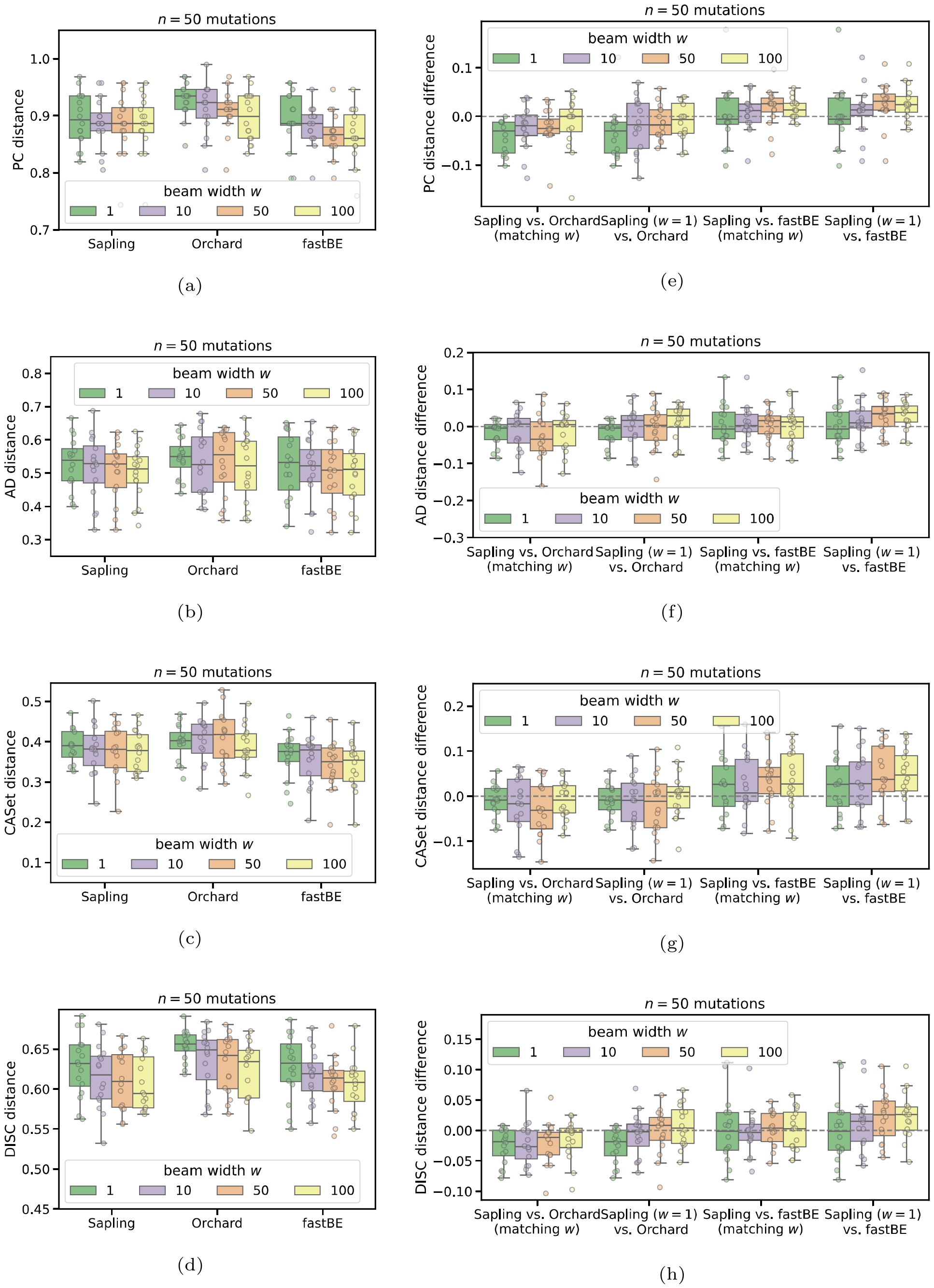


**Fig. S4**: **Inferring full trees on simulations with $n = 50$ mutations and $m = 10$ samples.** (a-d) Parent-child (PC), ancestor-descendant (AD), common ancestor set (CASet) and distinctly inherited set comparison (DISC) distances, respectively, of inferred to ground-truth trees for varying beam widths $w$. (e-f) Difference in PC, AD, CASet and DISC distances, respectively, between Sapling vs. Orchard and fastBE for matching instances and varying beam widths $w$. Negative values indicate Sapling inferred trees are closer to ground truth than the competing method.
